# Upregulation of Prickle2 Ameliorates Alzheimer’s Disease-Like Pathology in a Transgenic Mouse Model of Alzheimer’s Disease

**DOI:** 10.3389/fcell.2020.565020

**Published:** 2020-09-08

**Authors:** Fengxian Sun, Fang Jiang, Na Zhang, Hua Li, Weiping Tian, Weiying Liu

**Affiliations:** ^1^Department of Physiology and Pathophysiology, School of Basic Medical Sciences, Tianjin Medical University, Tianjin, China; ^2^Department of Cardiology, Tianjin Medical University General Hospital, Tianjin, China; ^3^Clinical College of Ophthalmology, Tianjin Eye Hospital, Tianjin Medical University, Tianjin, China; ^4^Research Center of Basic Medical Sciences, Tianjin Medical University, Tianjin, China; ^5^Department of Pathogen Biology, School of Basic Medical Sciences, Tianjin Medical University, Tianjin, China

**Keywords:** Prickle2, Alzheimer’s disease, oxidative stress, neuroinflammation, Wnt/PCP pathway

## Abstract

Alzheimer’s disease (AD) is a devastating neurodegenerative disorder that has no effective therapies. Prickle planar cell polarity protein 2 (Prickle2), is an important cytoplasmic regulator of Wnt/PCP signaling. It has been reported that Prickle2 deficiency reduced neurite outgrowth levels in mouse N2a cells and led to autism-like behaviors and hippocampal synaptic dysfunction in mice. However, much less is known about the relationship of Prickle2 to AD pathogenesis. RT-qPCR, Western blot and IHC results showed that the mRNA and protein levels of Prickle2 were reduced in APP/PS1/Tau transgenic (3xTg) mice. Intravenous injection of Prickle2-overexpressing AAV-PHP.eB vectors improved the cognitive deficits in 3xTg mice. We also demonstrated that Prickle2 could repress oxidative stress and neuroinflammation, ameliorate the amyloid β (Aβ) plaque pathology and reduce Tau hyperphosphorylation in APP/PS1 mice. Further investigation of the mechanism of Prickle2 in AD revealed that Prickle2 inhibited Wnt/PCP/JNK pathway in vivo and in vitro. Our results suggest that Prickle2 might be a potential candidate for the diagnosis and treatment of AD.

## Introduction

Alzheimer’s disease (AD) is a devastating neurodegenerative disorder with a high incidence rate among the elderly worldwide. It is characterized by progressive memory loss, cognitive dysfunctions and dementia (Whitaker et al., 2014). AD is mainly caused by the accumulation of extracellular amyloid β (Aβ) deposits, intracellular neurofibrillary tangles (NFTs) and loss of neurons and synapses (Oboudiyat et al., 2013; Thal et al., 2015; Nisbet and Gotz, 2018). Although many studies have investigated the etiology of AD in the last few decades, the molecular mechanism of this disease remains unclear, and effective therapy is still unsatisfactory (Barrera-Ocampo and Lopera, 2016; Agrawal et al., 2017; Suzuki et al., 2017; Cao et al., 2018; Panza et al., 2019). Therefore, it is urgent to unravel and understand the complex mechanisms of AD etiology in depth and to identify novel targets for AD diagnosis and treatment.

The Wnt/planar cell polarity (Wnt/PCP) signaling pathway, known as the non-canonical WNT signals independent β-catenin, was initially found to be involved in tissue polarity and cell movement (Veeman et al., 2003). Recently, Wnt/PCP signaling, which participates in development and a variety of human diseases (Katoh, 2005; Chen et al., 2018; Humphries and Mlodzik, 2018), is also associated with a higher risk of AD (Elliott et al., 2018; Sellers et al., 2018). Wnt/PCP signaling contains upstream canonical Wnt signaling pathway components, such as Wnt5A, Wnt5B, and Wnt11, for the transduction of Wnt/PCP signals, followed by several transmembrane protein receptors of the PCP core components, Frizzled (FZD), Dishevelled (DSH), Vangl (van Gogh-like) and the cytoplasmic regulator Prickle (Katoh, 2005; Barrow, 2006; VanderVorst et al., 2019). Most recently, intriguing studies have implicated the involvement of Prickle2 in the pathogenesis of nervous system diseases. For example, deletion of Prickle2 causes a lower seizure threshold and autism-like behaviors with hippocampal synaptic abnormalities (Tao et al., 2011; Ehaideb et al., 2014). Overexpression of Prickle2 promotes neurite outgrowth *in vitro* (Fujimura and Hatano, 2012). Accordingly, Prickle2 interacts with two autism spectrum disorder-related proteins, PSD-95 and the NMDA receptor in the postsynaptic density (Hida et al., 2011; Tao et al., 2011). Furthermore, the Prickle2 mRNA, predicted by an online tool AlzData (Xu et al., 2018), is markedly downregulated in the entorhinal cortex, temporal cortex and hippocampus of AD patients compared with that in normal people. However, the exact function of Prickle2 in the etiology of AD is poorly understood, and whether Prickle2 regulates Aβ generation and cognitive functions in AD is of interest.

In this study, we found that Prickle2 was downregulated in the brain tissue of 3 × Tg-AD mice, suggesting that the reduction of Prickle2 may play a certain role in the etiology of AD. AAV-mediated upregulation of Prickle2 ameliorates cognitive deficits and AD-like pathology in 3 × Tg transgenic mice by inhibiting the Wnt/PCP signaling pathway. Our results not only uncover the mechanism of Prickle2 in the etiology of AD but also provide a potential biomarker for the future diagnosis and treatment of AD.

## Materials and Methods

### Animals

The C57BL/6J mice (25–28 g; 6 months) were obtained and approved by Beijing HFK Bioscience Co., LET. APP/PS1/TAU (3 × Tg-AD; homozygous for the Psen1 mutation and homozygous for the co-injected APPSwe and tauP301L transgenes (Tg(APPSwe,tauP301L)1Lfa)) transgenic mice with the same background were obtained from the Jackson Laboratory, being created via co-microinjecting two independent transgenes encoding human APPswe and human TAUP301L into single-cell embryos harvested from homozygous mutant PS1m146v knockin (PS1-KI) mice. All animal procedures were approved by the Animal Care and Use Committee of Tianjin Medical University.

### Generation of AAV-PHP.eB Virus

The AAV-PHP.eB-Syn-mScarlet-P2A-MCS Rep-Cap trans plasmid was generated by gene synthesis (GenScript). Prickle2 (NM_001134461) was synthesized and cloned to AAV-PHP.eB -Syn-mScarlet-P2A-MCS to generate Prickle2 overexpressing vectors AAV-PHP.eB-Syn-mScarlet-P2A-MCS-Prickle2. Recom- binant AAVs were generated by triple transfection of HEK293T cells using polyethylenimine (PEI) and purified by ultracentrifugation over iodixanol gradients as previously described (Deverman et al., 2016).

### AAV-PHP.eB Vector Delivery

12–20 3 × Tg-AD or C57BL/6J (wild type, WT) mice were intravenously injected with AAV-PHP.eB vector and used for behavioral tests in each group. 3 × Tg+Prickle2 group was intravenously injected with AAV-PHP.eB-Syn-mScarlet-P2A-MCS-Prickle2 vectors, and the 3 × Tg control groups were intracerebrally injected with empty AAV-PHP.eB vector. Intravenous injection was performed in a restrainer that positioned the tail in a heated groove. The tail was swabbed with alcohol and then injected intravenously with a viral concentration 1 × 10^13^ vg/mL according to the experimental setup in a total volume of 100 μl of AAV-PHP.eB particles in PBS.

### Cell Culture and Cell Transfection

The mouse neuroblastoma N2a cell line was obtained from the American Tissue Culture Collection (Manassas, VA, United States). The cells were cultured in Dulbecco’s modified eagle medium (Sigma-Aldrich, Shanghai Trading Co. Ltd) containing 10% fetal bovine serum (FBS; Sigma-Aldrich, Shanghai Trading Co. Ltd) and 100 units penicillin-streptomycin (10,000 units/mL of penicillin and 10,000 μg/mL of streptomycin) at 37°C in a humidified atmosphere with 5% (v/v) CO_2_. N2a/APP695sw cell line was stably transfected with the human APP-695 Swedish mutation (K595N/M596L) and was maintained in a selective state by the antibiotic G-418 at a final concentration of 0.2 mg/ml in DMEM medium containing 10% FBS.

Cells were transfected using NeuroPorter^TM^ Transfection Kit (Sigma-Aldrich, United States) according to the manufacturer’s instructions. pcDNA3 (Vec), pSilencer 2.1-U6 Neo (shR-NC), pcDNA3-Prickle2 (Prickle2) and pSilencer-Prickle2 (shR-Prickle2) were constructed by GenePharma Co., Ltd. (Shanghai, China). Primers and oligonucleotides used for plasmid construction are listed in Supplementary Table S1.

### Real-Time Quantitative PCR

The total RNAs were extracted from brain tissues using TRIzol reagent (Ambion) according to the manufacturer’s instructions. Then, a NanoDrop ND-1000 spectrophotometer was used to quantify the isolated RNAs. After that, the cDNAs were prepared using the Thermo Scientific Revert Aid First Strand cDNA Synthesis Kit (Thermo Fisher Scientific, United States). RT-qPCR was performed using an SYBR Green Real-Time PCR Master Mix (Thermo Fisher Scientific, United States) on a Bio-Rad iQ5 system (Bio-Rad, Hercules, CA, United States). The relative expression levels of *Prickle2* were calculated by the 2^–ΔΔCt^ method as the endogenous controls. RT-qPCR primers are listed as follows (5′-3′): *Prickle2*: TGGAGAGAAGTTGCGAATCAAG (forward) and ACAAATAGCTCCTGTCATGGTG (reverse); *GAPDH*: CTGGGCTACACTGAGCACC (forward) and AAGTGGTCGTTGAGGGCAATG (reverse).

### Morris Water Maze

The behavioral test was performed as previously described using the Morris Water Maze (Jankowsky et al., 2007; Liu et al., 2017). Briefly, the navigation experiment was conducted on five consecutive days to record the time required for the mice to find the hidden platform. If the platform was not found, the mice were guided to the platform for 20 s, and the escape latency was recorded as 90 s. On day 6, the hidden platform was removed, and the mice were given a single 90 s to swim freely. The frequency of passing the hidden platform and the original angle were recorded.

### Tissue Collection

After the behavioral test, the mice were euthanised and then perfused with phosphate-buffered saline. Their brains were removed immediately. Part of their brain tissue was fixed in 4% paraformaldehyde solution, and then dehydrated, waxed and sectioned. For the remainder of the brain tissue, the hippocampus was separated from the cortex on an ice table, and then both were frozen in liquid nitrogen.

### Measurement of SOD, MDA and ROS

The hippocampus was separated from the cortex in the brain tissue, and then homogenized and centrifuged at 12000 × *g* for 15 min to isolate the supernatants, respectively. A malondialdehyde (MDA) assay kit (TBA method), superoxide dismutase (SOD) typed assay kit (hydroxylamine method), and reactive oxygen species assay kit (Nanjing Jiancheng Bioengineering Institute, China) were used to measure the MDA level, SOD activity and ROS level according to the manufacturer’s instructions, respectively.

### Immunohistochemical Staining

Serial 10 μm coronal sections were rehydrated, and antigen retrieval was performed with 0.01 M citric acid buffer for 20 min. The sections were then incubated with 3% H_2_O_2_ solution for 10 min to eliminate the activity of endogenous enzymes. Next, slides were incubated with blocking solutions (5% normal goat serum) at room temperature for 15 min and then incubated with the dilution of primary antibodies. The following primary antibodies were used: PRICKLE2 polyclonal antibody (Cat # PA5-96903, 1:100, Thermo Fisher Scientific, United States), Anti-Tau (phospho S396) antibody (Cat # ab109390, 1:1000, Abcam, United States). After incubation overnight at 4°C, the sections were incubated with a biotin-labeled secondary antibody for 15 min. Following the development, the sections were incubated with streptavidin-biotin complex and DAB peroxidase substrate kit was used to detect the expression of each protein. As a negative control, the sections were processed in the same step but used rabbit serum instead of primary antibodies. For Aβ immunofluorescence staining, brain paraffin section dewaxed in water, then the sections were incubated with Aβ4G8 mouse monoclonal antibody (cat # 800703, 1:100, Biolegend, United States), followed by goat anti-mouse IgG H&L (Alexa Fluor^®^ 488) (Cat # ab150113, 1:500, Abcam, United States). The tissue sections were also counterstained with DAPI, a nucleic acid-specific marker, to visualize the nuclei at a 633-nm excitation wavelength.

### X-34 and Thioflavin S (Thios) Staining

X-34 staining was performed according to previously described procedures (Styren et al., 2000). The brain paraffin sections were dewaxed in water, then the sections were incubated with 100 μM X-34 solution (dissolved in DMSO) and DAPI for 10 min and then further processed for immunohistochemistry. ThioS staining was performed according to previously described procedures (Wang et al., 2017). The brain paraffin section dewaxed in water, then the sections were incubated with 1% thioflavin S solution (dissolved in distilled water containing 50% ethanol) for 5 min and then differentiated in 50% ethanol for 5–15 min. Fluorescence imaging was collected using an Olympus fluorescence microscope (Olympus Corporation, Japan).

### ELISA

Aβ ELISA was performed according to the methods reported previously (Wang et al., 2017). Briefly, the cortical and hippocampal tissues were thoroughly homogenized using an animal tissue homogeniser. Then, the homogenates containing a protease inhibitor cocktail (Roche) were mixed 1:1 with 0.4% diethylamine (DEA). The mixture was centrifuged at 13,000 × *g* for 30 min at 4°C. The supernatants were isolated to measure soluble Aβ. In addition, the pellets were homogeneously mixed using 70% formic acid by sonication. Subsequently, the mixture was centrifuged at 13,000 × *g* for 30 min at 4°C, and the supernatants were collected to detect insoluble Aβ. Human Aβ40 or Aβ42 ELISA kits (Invitrogen) and IL-1β or IL-6 (Thermo Fisher Scientific) were used to measure the Aβ, IL-1β and IL-6 concentrations according to the manufacturer’s instructions.

### Western Blotting

The isolated hippocampus or cortex tissues were, respectively, mixed with RIPA buffer to extract protein, and the BCA method was used for the measurement of the total protein concentration. The proteins were separated by SDS–PAGE and transferred to PVDF membranes. The membranes were blocked with 5% skimmed milk for one hour at room temperature and then incubated with one of the primary antibodies overnight at 4°C. The following primary antibodies were used: PRICKLE2 polyclonal antibody (Cat # PA5-96903, 1:1000, Thermo Fisher Scientific, United States), Tau (phospho Ser356) antibody (Cat# ab75603, 1:1000, Abcam, United States), Tau (phospho S396) antibody (Cat # ab109390, 1:5000, Abcam, United States), Tau (phospho S202) antibody (Cat # ab108387, 1:5000, Abcam, United States), JNK antibody (Cat # ab179461, 1:500, Abcam, United States), JNK (phospho T183 + Y185) antibody (Cat# ab4821, 1:500, Abcam, United States), c-Jun antibody (Cat # 9165, 1:500, Cell Signaling Technology, United States), c-Jun (phospho Ser63) antibody (Cat # 9261, 1:500, Cell Signaling Technology, United States), GAPDH antibody (Cat # 5174, 1:1000, Cell Signaling Technology, United States). Next, the membranes were washed and incubated with HRP-conjugated secondary antibody for two hours. Then, the ECL detection was used to visualize the bands, which were then quantified using the ImageJ analysis tool.

### Statistical Analysis

Statistical analysis was performed using GraphPad Prism8 software. All values were reported as mean ± SEM. A Student’s *t* test or ANOVA, followed by Bonferroni’s post hoc test, were used to analyze the differences between groups. A *p-*value < 0.05 was considered statistically significant.

## Results

### Prickle2 Was Dysregulated in AD

**FIGURE 1:**
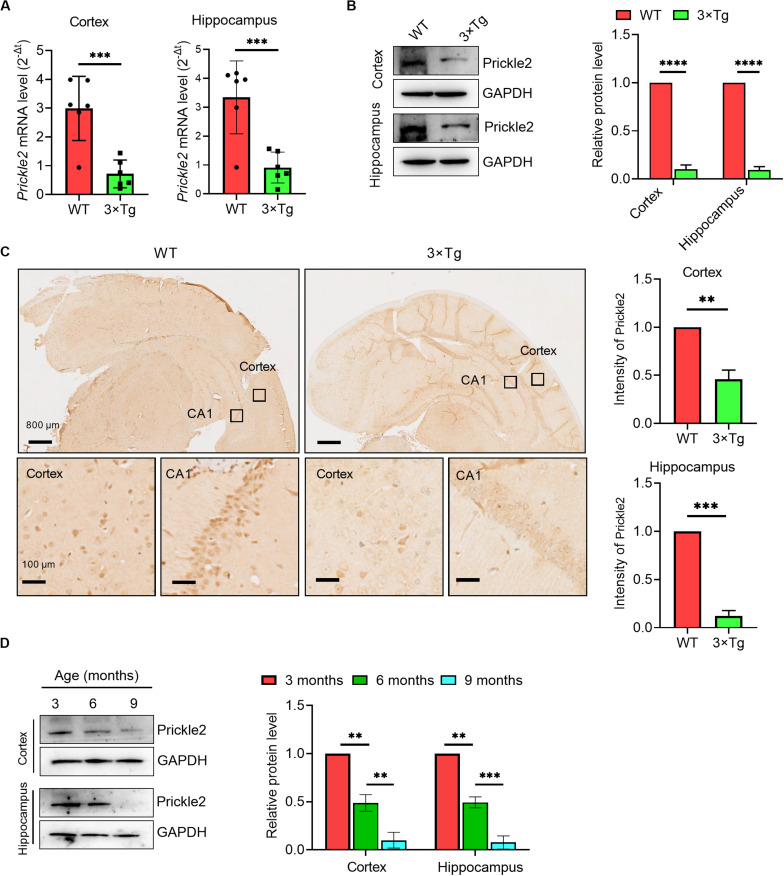
Prickle2 is downregulated in the brain tissues of 3 × Tg mice. **(A)** *Prickle2* mRNA (*n* = 6 per group) in the cortex and the hippocampus isolated from mouse brain. **(B)** Western blotting for Prickle2 in the cortex and the hippocampus isolated from mouse brain (*n* = 6 per group). **(C)** IHC for Prickle2 in cortex and hippocampus isolated from mouse brain (*n* = 6 per group). **(D)** Western blots in cell lysates from AD mice during aging with antibodies against Prickle2 and GAPDH, as indicated. Blotting intensities are normalized to GAPDH from AD mice at three months old of age (defined as 1.0, *n* = 6) **(A–D)** Student’s *t* test. ^∗∗^*p* < 0.01; ^∗∗∗^*p* < 0.001; ^*⁣*⁣**^p < 0.0001.

To investigate the role of Prickle2 in AD, we detected the transcription and expression of Prickle2 in a model of AD using 3 × Tg mice by RT-qPCR, Western blot and IHC, respectively. The results showed that Prickle2 was significantly downregulated in the cortex and the hippocampus of 3 × Tg mice compared with WT mice (Figures 1A–C). We then analyzed the expression of Prickle2 in 3 × Tg mice during aging by Western blot. The results showed that Prickle2 expression gradually decreased in 3 × Tg mice from when they were three months old to 9 months old (Figure 1D). Collectedly, these results indicated that the downregulation of Prickle2 in 3 × Tg-AD mouse model might be related to AD etiology.

### Prickle2 Improved the Cognitive Deficits in 3 × Tg-AD Mice

**FIGURE 2:**
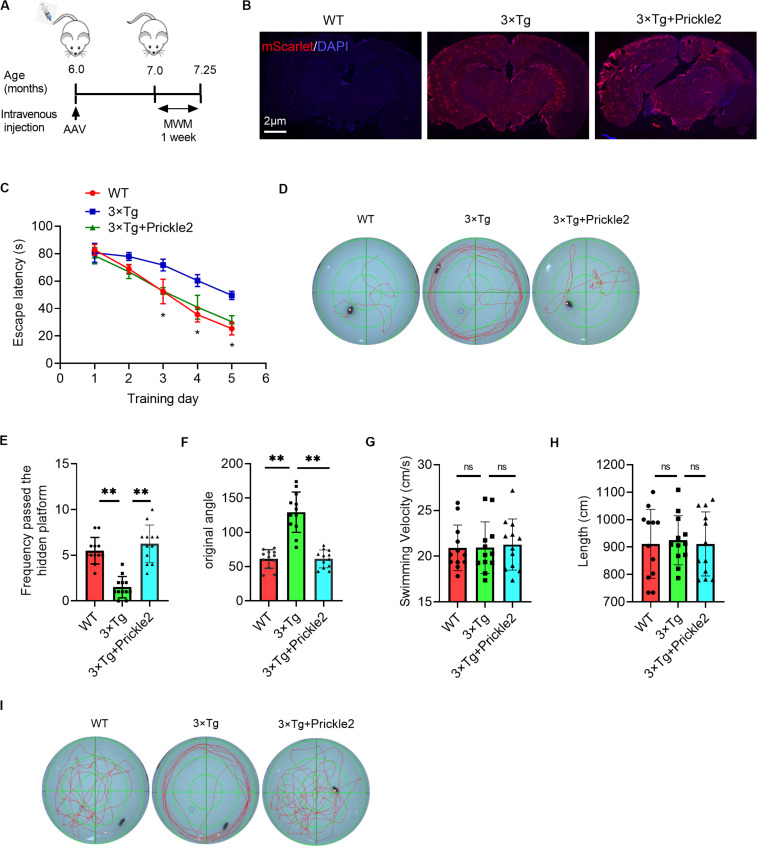
AAV-mediated Prickle2 overexpression ameliorates impaired learning and memory in 3 × Tg mice. **(A)** Experimental schedule for AAV-PHP.eB-mediated Prickle2 treatment. **(B)** Evaluation of the infection efficiency after injection of AAV-PHP.eB virus. Immunofluorescence analysis showing the expression of mScarlet in the brain coronal sections. **(C)** Escape latency plotted according to the training days. **(D)** Typical swimming traces during the escape latency process through MWM test on day 5. **(E)** Frequency passed the hidden platform and **(F)** original angle data plotted during the MWM test on day 6. **(G)** The swim velocity and length **(H)** was calculated, and there was no significant group difference in average swim velocity or length. **(I)** Typical spatial probe process during the MWM test on day 6. All experiments were performed on 7-month-old mice (*n* = 12). ANOVA followed by Bonferroni’s post hoc test. ^∗^*p* < 0.05; ^∗∗^*p* < 0.01; ns, not significant.

The downregulation of Prickle2 in 3 × Tg mice led us to evaluate the role of Prickle2 by the tail intravenous injection of Prickle2 overexpressing AAV-PHP.eB vectors (AAV-PHP.eB-Syn-mScarlet-P2A-MCS-Prickle2) and control AAV-PHP.eB vectors (AAV-PHP.eB-Syn-mScarlet-P2A-MCS) into 6-month-old 3 × Tg transgenic mice (Figure 2A). We then assessed the distribution of viruses in the brain at three weeks post-injection, using immunofluorescence to confirm that the delivery was effective. Moreover, the neurons of the cortex and the hippocampus in 3 × Tg mice were infected by AAV-PHP.eB (Figure 2B). This confirmation indicated that AAV-PHP.eB vectors were able to effectively deliver in brain cells in mice after tail intravenous injection. Then, we tested the effect of Prickle2 on the learning and memory deficits in 3 × Tg mice using the MWM. The escape latency test results suggested that treatment with AAV-PHP.eB-Syn-mScarlet-P2A-MCS-Prickle2, but not AAV-PHP.eB-Syn-mScarlet-P2A-MCS (control), exerted a markedly shorter escape latency on day 5 (Figures 2C,D). In addition, the frequency passed the hidden platform for Prickle2-overexpressed 3 × Tg mice was higher than 3 × Tg mice on day 6 (Figure 2E). Finally, the spatial probe test (with no platform) results showed that Prickle2-overexpressed 3 × Tg mice displayed an increased target quadrant occupation compared with the 3 × Tg mice, indicating that the Prickle2-overexpressed 3 × Tg mice had a good ability to find the platform compared with the 3 × Tg mice (Figures 2F–I). The data demonstrated that overexpression of Prickle2 could improve cognitive deficits in 3 × Tg mice.

### Prickle2 Reduced Oxidative Stress and Inflammation in the Brains of 3 × Tg Mice

**FIGURE 3:**
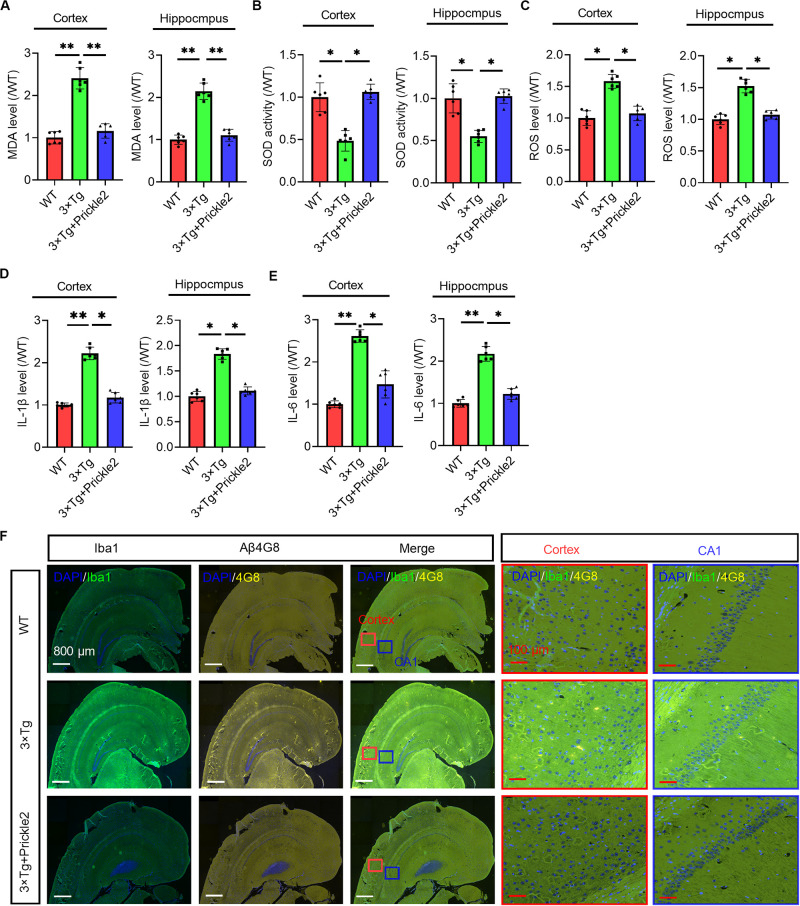
Prickle2 overexpression ameliorates oxidative stress and neuroinflammation in 3 × Tg mice. **(A)** The level of MDA and **(B)** the activity of SOD was determined in the brain of WT, 3 × Tg and 3 × Tg/Prickle2 treatment mice (*n* = 6 per group). **(C)** The ROS level was measured in the supernatants of the brain homogenates of WT, 3 × Tg and 3 × Tg/Prickle2 treatment mice (*n* = 6 per group). **(D)** The levels of IL-1β and **(E)** IL-6 were determined by ELISA in the brain of WT, 3 × Tg and 3 × Tg/Prickle2 treatment mice (*n* = 6 per group). **(F)** Brain sections of the cortex and hippocampus were stained with anti-Iba1 and anti-Aβ4G8 antibodies. **(A–E)** ANOVA followed by Bonferroni’s post hoc test. ^∗^*p* < 0.05; ^∗∗^*p* < 0.01.

In the pathogenesis of AD, neural oxidative stress and neuroinflammation could lead to the progressive impairment of cognition. Therefore, we measured the level of MDA, the activity of SOD and the level of ROS. The results showed that the levels of MDA and ROS were markedly enhanced and the activity of SOD was significantly reduced in the brains of 3 × Tg mice compared with those of WT mice, however. Prickle2 overexpression markedly relieved the oxidative stress status (Figures 3A–C). Accordingly, we observed that two key neuroinflammatory markers, IL-1β and IL-6, were noticeably enhanced in the brains of 3 × Tg mice compared with those of WT mice (Figures 3D,E), whereas the upregulation of Prickle2 significantly inhibited the levels of IL-1β and IL-6 in 3 × Tg mice. It is well known that the activation of microglia or astrocytes is an important component in neuroinflammation mediated AD pathology. Herein, we examined whether the upregulation of Prickle2 could inhibit microglia activation in 3 × Tg mice. Coronal sections of the brain tissues were stained with an antibody against Iba1, which is a biomarker of microglia activation. We showed that the upregulation of Prickle2 displayed less intense immunohistochemical Iba1 labeling in the cortex and hippocampus than did vehicle treated 3 × Tg mice (Figure 3F). These results indicated that Prickle2 overexpression could repress oxidative stress and neuroinflammation in 3 × Tg mice.

### Prickle2 Reduced Amyloid Plaque Pathology in the Brains of 3 × Tg Mice

**FIGURE 4:**
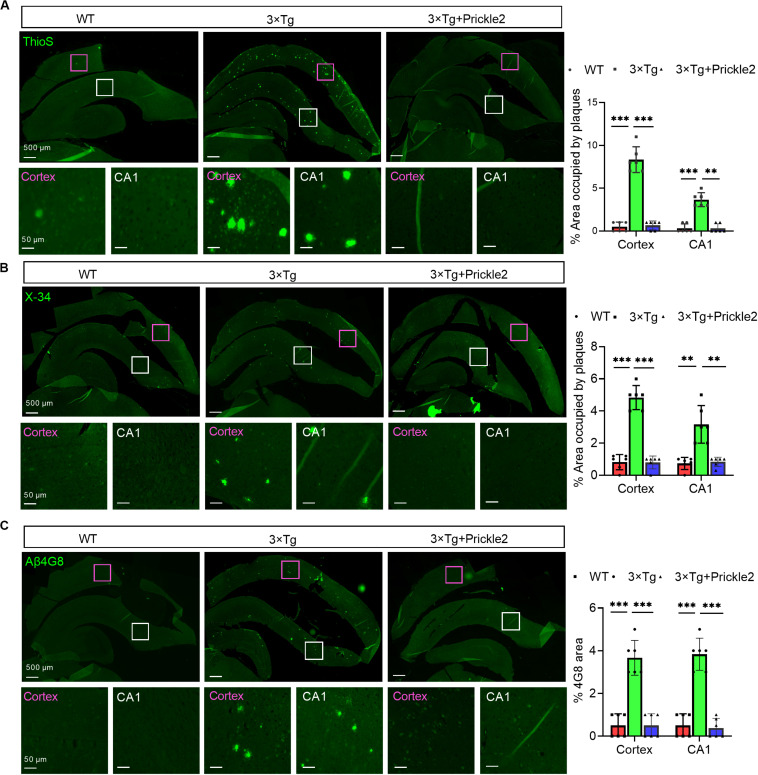
Prickle2 treatment diminishes Aβ deposition and Aβ levels in 3 × TG mice. **(A)** Representative thioflavin S (ThioS, Aβ plaques) immunofluorescence images in cortex and hippocampus of WT, 3 × Tg and 3 × Tg/Prickle2 treatment mice. The percentage of cortical and hippocampal area occupied by Aβ plaques (*n* = 6 per group). **(B)** Representative images of WT, 3 × Tg and 3 × Tg/Prickle2 treatment mice stained with X-34 and the percentage of cortical and hippocampal area stained with X-34 was quantified (*n* = 6 per group). **(C)** Representative immunofluorescence images stained byβ-Amyloid 4G8 antibody. The percentage of cortical and hippocampal area stained withβ-Amyloid 4G8 antibody was quantified (*n* = 6 per group). ANOVA followed by Bonferroni’s post hoc test. ^∗∗^*p* < 0.01; ^∗∗∗^*p* < 0.001.

First, we measured the Aβ profile to determine whether Prickle2 affected the deposition of Aβ in 3 × Tg mice. The results of thioflavin S (ThioS) staining showed that Prickle2-overexpressed 3 × Tg mice displayed fewer mature amyloid plaques than vehicle-treated 3 × Tg mice (Figure 4A). Then, we found that the fibrillar amyloid burden was significantly reduced by Prickle2-overexpressed 3 × Tg mice using X-34, which has been proven to label amyloid structures (Figure 4B). In addition, we used β-Amyloid (4G8) immunostaining for detection of total amyloid plaques, which showed a significant decrease in Prickle2-overexpressed 3 × Tg mice compared with control 3 × Tg mice (Figure 4C). Moreover, we performed ELISA to measure several Aβ isoforms in the cortex and the hippocampus. Both soluble and insoluble Aβ40 and Aβ42 levels in the brains of Prickle2-overexpressed 3 × Tg mice were significantly decreased compared with vehicle-treated 3 × Tg mice (Supplementary Figures S1A,B). These results indicated that the upregulation of Prickle2 ameliorated amyloid plaque pathology in the brains of 3 × T1 mice.

### Prickle2 Ameliorated Tau Phosphorylation in the Brains of 3 × Tg Mice

**FIGURE 5:**
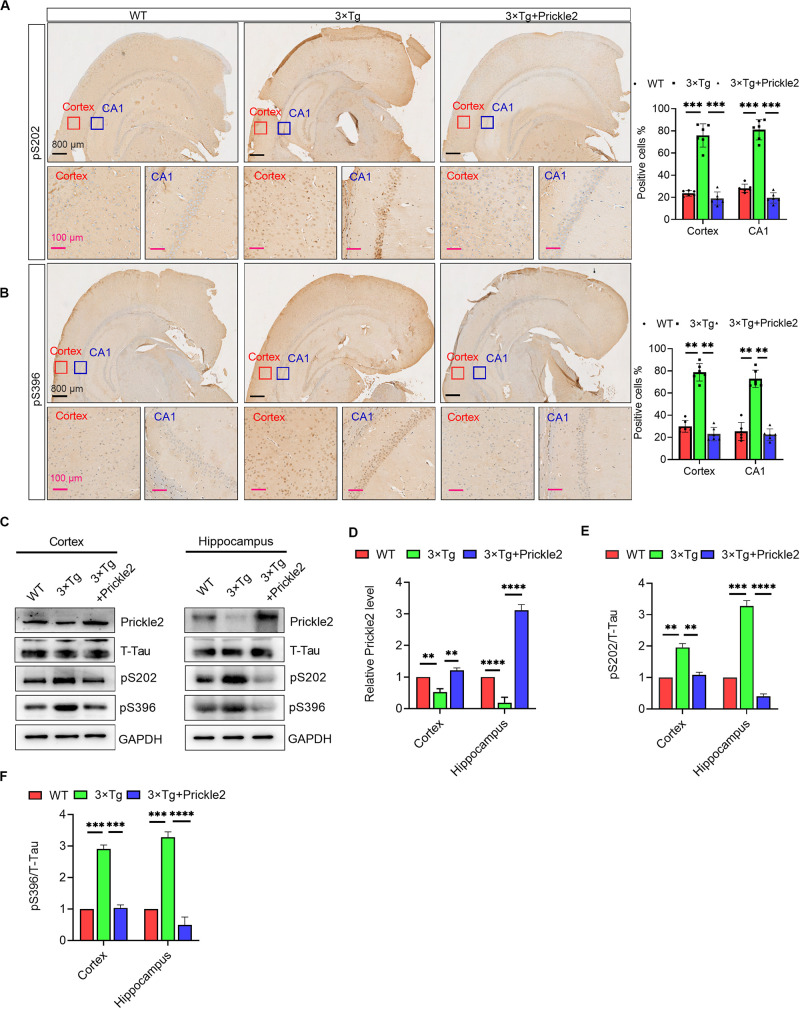
Prickle2 treatment reduces Tau phosphorylation in 3 × Tg mice. **(A)** Representative IHC images of WT, 3 × Tg and 3 × Tg/Prickle2 treatment mice stained with anti-Tau (phospho S202) antibodies. The proportions of pS202-positive cells were calculated in cortical and hippocampal, respectively (*n* = 6 per group). **(B)** Representative IHC images of WT, 3 × Tg and 3 × Tg/Prickle2 treatment mice stained with anti-Tau (phospho S396) antibodies. The proportions of pS396-positive cells were calculated in cortical and hippocampal, respectively (*n* = 6 per group). **(C)** Western blots detected the phosphorylated Tau using pS202 and pS396 antibodies in the brain tissues from WT, 3 × Tg and 3 × Tg/Prickle2 mice. **(D–F)** Quantification of the protein levels of phosphorylated Tau by densitometric analyses (*n* = 6 per group). **(B–D)** ANOVA followed by Bonferroni’s post hoc test. ^∗∗^*p* < 0.01; ^∗∗∗^*p* < 0.001; ^*⁣*⁣**^p < 0.0001.

IHC staining of the cortex and the hippocampus was performed using antibodies against phospho-Tau S202 and S396 to confirm whether upregulation of Prickle2 could affect Tau pathology in 3 × Tg mice. The results of IHC staining showed that phospho-Tau S202 and pS396 protein levels in the cortex and the hippocampus were markedly reduced in Prickle2-overexpressed 3 × Tg mice than in control 3 × Tg mice (Figures 5A,B). Consistently, Western blots results showed that the protein levels of Prickle2 in the cortex and hippocampus were markedly reduced in Prickle2-overexpressed 3 × Tg mice compared with control 3 × Tg mice. In addition, phospho-Tau S202 and phospho-Tau S396 protein levels in the cortex and hippocampus were markedly reduced in Prickle2-overexpressed 3 × Tg mice compared with control 3 × Tg mice (Figures 5C–F). These results suggested that Prickle2 overexpression reduced Tau hyperphosphorylation in the brains of 3 × Tg mice.

### Prickle2 Ameliorated AD-Like Pathology via Inhibition of the Wnt/PCP Pathway

**FIGURE 6:**
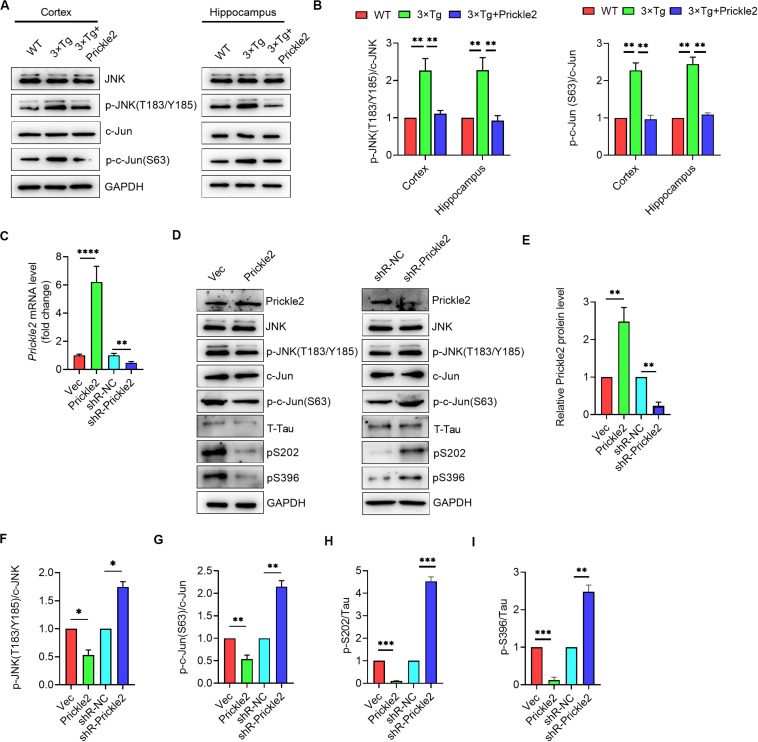
Prickle2 treatment ameliorates AD-like pathology through Wnt/PCP pathway. **(A)** Western blotting analysis of protein levels of JNK, p-JNK (T183/Y185), c-Jun, and p-c-Jun (S63) in brain tissues from WT, 3 × TG and 3 × TG/Prickle2 treatment mice (*n* = 6 per group). **(B)** Quantification of the protein levels of p-JNK (T183/Y185) and p-c-Jun (S63) by densitometric analyses (*n* = 6 per group). **(C)** *Prickle2* mRNA (*n* = 3 per group) in N2a/APP695sw cells transfected with empty vector pcDNA3 (Vec), pcDNA3-Prickle2, pSilencer 2.1-U6 neo (shR-NC), and pSilencer-Prickle2 (shR-Prickle2). **(D)** Western blotting analysis of protein levels of Prickle2, JNK, p-JNK (T183/Y185), c-Jun, p-c-Jun (S63), Tau, p-Tau S202 and p-Tau S396 in N2a/APP695sw cells transfected with indicated plasmids or siRNAs. **(E–I)** Quantification of the protein levels of Prickle2, p-JNK (T183/Y185), p-c-Jun (S63) p-Tau S202 and p-Tau S396 by densitometric analyses (*n* = 3 per group). **(B,C,E–I)** ANOVA followed by Bonferroni’s post hoc test. ^∗^*p* < 0.05; ^∗∗^*p* < 0.01; ^∗∗∗^*p* < 0.001.

Prickle2 acts on a core component of the Wnt/PCP pathway, which has shown to transduce signals mainly via the JNK-mediated signaling pathway in AD (Killick et al., 2014). We further investigated the potential mechanisms responsible for the effect of Prickle2 on AD etiology. We detected the protein levels of JNK, p-JNK (T183/Y185), c-Jun and p-c-Jun (S63) in brain tissues from WT, 3 × Tg and 3 × Tg/Prickle2-treated mice by Western blot. Results showed that Prickle2 overexpression markedly reduced the levels of p-JNK (T183/Y185) and p-c-Jun (S63) (Figures 6A,B). We further confirmed the mechanisms responsible for the effect of Prickle2 on the etiology of AD by transfection with an empty vector (Vec), pcDNA3/prickle2, si-NC and si-Prickle2 to a cell model of AD (N2a/APP695sw) by stably expressed human APP-695 Swedish mutation (K595N/M596L) in N2a cells. RT-qPCR was performed to detect the transcriptional level of *Prickle2*, and results showed that the level of *Prickle2* mRNA markedly increased after transfection with pcDNA3/Prickle2, whereas the level of *Prickle2* mRNA markedly decreased after transfection with si-Prickle2 in N2a/APP695sw cells (Figure 6C). In addition, we detected the protein levels of Prickle2, JNK, p-JNK (T183/Y185), c-Jun, p-c-Jun (S63), phospho-Tau S202 and pS396 by Western blot. Results showed that Prickle2 overexpression markedly decreased the levels of p-JNK (T183/Y185), p-c-Jun (S63), phospho-Tau S202 and pS396, whereas the silence of Prickle2 markedly increased the levels of p-JNK (T183/Y185), p-c-Jun (S63), phospho-Tau S202 and pS396 in N2a/APP695sw cells (Figures 6D–I). Taken together, these results suggested that Prickle2 overexpression alleviated AD-like neurodegeneration via inhibiting the Wnt/PCP pathway.

## Discussion

Alzheimer’s disease is a complex and progressive cognitive dysfunction disease. Although many molecules have been proven to be involved in the pathogenesis of AD, the etiology of AD remains unclear. Recent studies have shown that the Wnt/PCP pathway contributed to the development and several human diseases (Yang, 2012; Zhang et al., 2017; Humphries and Mlodzik, 2018; Lopez-Escobar et al., 2018; Sellers et al., 2018). Therefore, further investigation of specific facets of the Wnt/PCP pathway might confer certain advantages to improve our understanding regarding the diagnosis and treatment of AD in the future.

To our best knowledge, Wnts are known as ligands for FZD receptors of PCP signaling (Bhanot et al., 1996; Wang et al., 2006). Recently, Wnt5a was demonstrated to interact genetically with Ltap/Vangl2 ad functioned in the regulation of PCP in mice (Qian et al., 2007). Tang et al. reported that Wnt5a overexpression promoted Aβ-evoked inflammatory responses in AD-related neurodegeneration during AD pathogenesis (Li et al., 2011). In addition, APP was demonstrated to activate Wnt/PCP signaling by physical interaction with the Wnt co-receptor VANGL2 to promote synapse loss and Aβ production (Elliott et al., 2018). Moreover, Disheveled 1, a member of the PCP core components, has been demonstrated to increase sAPPa and reduce Tau phosphorylation via both JNK and protein kinase C (PKC)/mitogen-activated protein kinase signaling *in vitro* (Mudher et al., 2001). Taken together, several core components of Wnt/PCP signaling were demonstrated to be involved in the pathogenesis of AD.

Prickle2, the cytoplasmic regulator of Wnt/PCP signaling, has been reported to localize with Frizzled and Disheveled proteins (Katoh and Katoh, 2003). Okuda et al. (2007) have illustrated that the loss of Prickle2 could markedly reduce the neurite outgrowth levels in mouse N2a cells. Furthermore, Prickle2 was demonstrated to interact with PSD-95 and NMDA receptors and localize in the postsynaptic density in the mice brain (Hida et al., 2011). In addition, (Lopez-Escobar et al., 2018) have demonstrated that the Prickle2 deletion could cause autism-like behaviors and hippocampal synaptic dysfunction in mice. Moreover, (Sowers et al., 2013) found that morphological deficits caused by the Prickle2 deletion in hippocampal neurons could be rescued by the non-canonical Wnt ligand Wnt5a, which has been demonstrated to be involved in the pathogenesis of AD. Although several studies have demonstrated that Prickle2 could be related to the nervous system, the exact function of this gene in the etiology of AD was unreported.

Herein, we first reported that the *Prickle2* mRNA level was significantly downregulated in the cortex and the hippocampus of 3 × Tg mice, indicating that Prickle2 might be related to the etiology of AD. Subsequently, we upregulated the expression level of Prickle2 in the brains of 3 × Tg mice by intracerebral injection of Prickle2-overexpressing AAV9 vectors and found that Prickle2 could improve the cognitive deficits in 3 × Tg mice. In addition, we found that the upregulation of Prickle2 inhibited oxidative stress, neuroinflammation, amyloid plaque pathology and Tau hyperphosphorylation in the brains of 3 × Tg mice. It was well known that Aβ accumulation increased the superoxide anion, MDA and ROS. However, it also inhibited the activity of catalase and SOD, aggravated oxidative damage and neuroinflammation in the brain with AD (Cai et al., 2014; Tonnies and Trushina, 2017). In our study, we showed that the contents of MDA and ROS were markedly enhanced, and the activity of SOD was greatly reduced in 3 × Tg mice, whereas Prickle2 overexpression relieved the oxidative stress. IL-1β and IL-6, two key proinflammatory factors that could also exacerbate AD pathology, have been reported to be increased by Aβ accumulation in 3 × Tg mice (de Lemos et al., 2013; Hoeijmakers et al., 2017). Our study found that Prickle2 overexpression reduced the levels of IL-1β and IL-6 in 3 × Tg mice. Taken together, the above results indicated that Prickle2 could ameliorate AD-like pathology in 3 × Tg mice.

We further investigated the potential mechanisms responsible for the effect of Prickle2 on the etiology of AD. We found that Prickle2 overexpression alleviated AD-like neurodegeneration via the inhibition of the Wnt/PCP/JNK pathway *in vivo* and *in vitro*. Activation of the JNK signaling pathway has been well known as a critical regulator for neurodegenerative diseases, plaque formation, neuroinflammation and maturation in the pathogenesis of AD (Yarza et al., 2015; Zhu et al., 2019). In addition to Wnt/PCP pathway, numerous studies have shown that a sustained loss of function of Wnt/β- catenin signaling components underlies the onset and progression of the AD (De Ferrari et al., 2014). Activation of Wnt-β-catenin signaling could stabilize synapses and increase the formation of new ones, while Wnt-PCP signaling could destabilize synapses and enhance the loss of synaptic connections (Elliott et al., 2018; Palomer et al., 2019). Mattes et al. (2018) reported that Wnt/PCP signaling could control the spreading of Wnt/β-catenin signals by cytonemes in vertebrates. In summary, a number of studies strongly suggests that restoring or boosting Wnt signaling could protect cells and synapses from Aß toxicity and ameliorate AD pathology.

In summary, we first demonstrated that Prickle2 was downregulated in the cortex and the hippocampus of 3 × Tg mice. The role of Prickle2 in the pathogenesis of AD was demonstrated by tail intravenous injection of Prickle2-overexpressing AAV-PHP.eB vectors. We found that Prickle2 could improve the cognitive deficits in 3 × Tg mice by ameliorating the oxidative stress, neuroinflammation, amyloid plaque pathology and Tau hyperphosphorylation. Further investigation of the mechanism of Prickle2 in AD revealed that Prickle2 could inhibit the Wnt/PCP/JNK pathway *in vivo* and *in vitro* Our results suggested that Prickle2 holds potential as a promising candidate for the diagnosis and treatment of AD.

## Data Availability Statement

All datasets presented in this study are included in the article/Supplementary Material.

## Ethics Statement

The animal study was reviewed and approved by Animal Care and Use Committee of Tianjin Medical University. Written informed consent was obtained from the owners for the participation of their animals in this study.

## Author Contributions

WL conceived the study. WL, FS, FJ, and WT designed all figures and wrote the manuscript. FS and FJ performed the experiments. NZ, HL, and WT performed the data analysis. All authors reviewed the manuscript and approved its final version.

## Conflict of Interest

The authors declare that the research was conducted in the absence of any commercial or financial relationships that could be construed as a potential conflict of interest.

## References

[B1] Agrawal M. Ajazuddin, Tripathi D. K. Saraf S. Antimisiaris S. G. Mourtas S. (2017). Recent advancements in liposomes targeting strategies to cross blood-brain barrier (BBB) for the treatment of Alzheimer’s disease. *J. Control Release* 260 61–77. 28549949 10.1016/j.jconrel.2017.05.019

[B2] Barrera-Ocampo A. Lopera F. (2016). Amyloid-beta immunotherapy: the hope for Alzheimer disease? *Colomb. Med.* 47 203–212. 10.25100/cm.v47i4.2640 28293044 PMC5335861

[B3] Barrow J. R. (2006). Wnt/PCP signaling: a veritable polar star in establishing patterns of polarity in embryonic tissues. *Semin. Cell Dev. Biol.* 17 185–193. 10.1016/j.semcdb.2006.04.002 16765615

[B4] Bhanot P. Brink M. Samos C. H. Hsieh J. C. Wang Y. Macke J. P. (1996). A new member of the frizzled family from Drosophila functions as a Wingless receptor. *Nature* 382 225–230. 10.1038/382225a0 8717036

[B5] Cai Z. Hussain M. D. Yan L. J. (2014). Microglia, neuroinflammation, and beta-amyloid protein in Alzheimer’s disease. *Int. J. Neurosci.* 124 307–321. 10.3109/00207454.2013.833510 23930978

[B6] Cao J. Hou J. Ping J. Cai D. (2018). Advances in developing novel therapeutic strategies for Alzheimer’s disease. *Mol. Neurodegener.* 13 018–0299. 10.1186/s13024-018-0299-8 PMC6291983 30541602

[B7] Chen Z. Lei Y. Cao X. Zheng Y. Wang F. Bao Y. (2018). Genetic analysis of Wnt/PCP genes in neural tube defects. *BMC Med. Genomics* 11:38. 10.1186/s12920-018-0355-9 29618362 PMC5885375

[B8] De Ferrari G. V. Avila M. E. Medina M. A. Perez-Palma E. Bustos B. I. Alarcon M. A. (2014). Wnt/β-catenin signaling in Alzheimer’s disease. *CNS Neurol. Disord. Drug Targets* 13 745–754. 24365184 10.2174/1871527312666131223113900

[B9] de Lemos M. L. De La Torre A. V. Petrov D. Brox S. Folch J. Pallas M. (2013). Evaluation of hypoxia inducible factor expression in inflammatory and neurodegenerative brain models. *Int. J. Biochem. Cell Biol.* 45 1377–1388. 10.1016/j.biocel.2013.04.011 23603149

[B10] Deverman B. E. Pravdo P. L. Simpson B. P. Kumar S. R. Chan K. Y. Banerjee A. (2016). Cre-dependent selection yields AAV variants for widespread gene transfer to the adult brain. *Nat. Biotechnol.* 34 204–209. 10.1038/nbt.3440 26829320 PMC5088052

[B11] Ehaideb S. N. Iyengar A. Ueda A. Iacobucci G. J. Cranston C. Bassuk A. G. (2014). Prickle modulates microtubule polarity and axonal transport to ameliorate seizures in flies. *Proc. Natl. Acad. Sci. U.S.A.* 111 11187–11192. 10.1073/pnas.1403357111 25024231 PMC4121842

[B12] Elliott C. Rojo A. I. Ribe E. Broadstock M. Xia W. Morin P. (2018). A role for APP in Wnt signalling links synapse loss with beta-amyloid production. *Transl. Psychiatry* 8 018–0231. 10.1038/s41398-018-0231-6 PMC6145937 30232325

[B13] Fujimura L. Hatano M. (2012). Role of Prickle1 and Prickle2 in neurite outgrowth in murine neuroblastoma cells. *Methods Mol. Biol.* 839 173–185. 10.1007/978-1-61779-510-7_14 22218901

[B14] Hida Y. Fukaya M. Hagiwara A. Deguchi-Tawarada M. Yoshioka T. Kitajima I. (2011). Prickle2 is localized in the postsynaptic density and interacts with PSD-95 and NMDA receptors in the brain. *J. Biochem.* 149 693–700. 10.1093/jb/mvr023 21324980

[B15] Hoeijmakers L. Ruigrok S. R. Amelianchik A. Ivan D. Van Dam A. M. Lucassen P. J. (2017). Early-life stress lastingly alters the neuroinflammatory response to amyloid pathology in an Alzheimer’s disease mouse model. *Brain Behav. Immun.* 63 160–175. 10.1016/j.bbi.2016.12.023 28027926

[B16] Humphries A. C. Mlodzik M. (2018). From instruction to output: Wnt/PCP signaling in development and cancer. *Curr. Opin. Cell Biol.* 51 110–116. 10.1016/j.ceb.2017.12.005 29289896 PMC5949250

[B17] Jankowsky J. L. Younkin L. H. Gonzales V. Fadale D. J. Slunt H. H. Lester H. A. (2007). Rodent A beta modulates the solubility and distribution of amyloid deposits in transgenic mice. *J. Biol. Chem.* 282 22707–22720. 10.1074/jbc.m611050200 17556372 PMC4435736

[B18] Katoh M. (2005). WNT/PCP signaling pathway and human cancer (review). *Oncol. Rep.* 14 1583–1588. 16273260

[B19] Katoh M. Katoh M. (2003). Identification and characterization of human PRICKLE1 and PRICKLE2 genes as well as mouse Prickle1 and Prickle2 genes homologous to Drosophila tissue polarity gene prickle. *Int. J. Mol. Med.* 11 249–256. 12525887

[B20] Killick R. Ribe E. M. Al-Shawi R. Malik B. Hooper C. Fernandes C. (2014). Clusterin regulates β-amyloid toxicity via Dickkopf-1-driven induction of the wnt-PCP-JNK pathway. *Mol. Psychiatry* 19 88–98. 10.1038/mp.2012.163 23164821 PMC3873038

[B21] Li B. Zhong L. Yang X. Andersson T. Huang M. Tang S. J. (2011). WNT5A signaling contributes to Abeta-induced neuroinflammation and neurotoxicity. *PLoS One* 6:e22920. 10.1371/journal.pone.0022920 21857966 PMC3157339

[B22] Liu W. Sun F. Wan M. Jiang F. Bo X. Lin L. (2017). beta-Sheet Breaker Peptide-HPYD for the treatment of Alzheimer’s disease: primary studies on behavioral test and transcriptional profiling. *Front. Pharmacol.* 8:969. 10.3389/fphar.2017.00969 29358920 PMC5766670

[B23] Lopez-Escobar B. Caro-Vega J. M. Vijayraghavan D. S. Plageman T. F. Sanchez-Alcazar J. A. Moreno R. C. (2018). The non-canonical Wnt-PCP pathway shapes the mouse caudal neural plate. *Development* 145:dev157487. 10.1242/dev.157487 29636380 PMC5992595

[B24] Mattes B. Dang Y. Greicius G. Kaufmann L. T. Prunsche B. Rosenbauer J. (2018). Wnt/PCP controls spreading of Wnt/β-catenin signals by cytonemes in vertebrates. *eLife* 7:e36953. 10.7554/eLife.36953 PMC6086664 30060804

[B25] Mudher A. Chapman S. Richardson J. Asuni A. Gibb G. Pollard C. (2001). Dishevelled regulates the metabolism of amyloid precursor protein via protein kinase C/mitogen-activated protein kinase and c-Jun terminal kinase. *J. Neurosci.* 21 4987–4995. 10.1523/jneurosci.21-14-04987.2001 11438574 PMC6762860

[B26] Nisbet R. M. Gotz J. (2018). Amyloid-beta and Tau in Alzheimer’s disease: novel pathomechanisms and non-pharmacological treatment strategies. *J. Alzheimers Dis.* 64 S517–S527. 29562514 10.3233/JAD-179907

[B27] Oboudiyat C. Glazer H. Seifan A. Greer C. Isaacson R. S. (2013). Alzheimer’s disease. *Semin. Neurol.* 33 313–329. 24234352 10.1055/s-0033-1359319

[B28] Okuda H. Miyata S. Mori Y. Tohyama M. (2007). Mouse Prickle1 and Prickle2 are expressed in postmitotic neurons and promote neurite outgrowth. *FEBS Lett.* 581 4754–4760. 10.1016/j.febslet.2007.08.075 17868671

[B29] Palomer E. Buechler J. Salinas P. C. (2019). Wnt signaling deregulation in the aging and Alzheimer’s brain. *Front. Cell Neurosci.* 13:227. 10.3389/fncel.2019.00227 31191253 PMC6538920

[B30] Panza F. Lozupone M. Seripa D. Imbimbo B. P. (2019). Amyloid-beta immunotherapy for alzheimer disease: is it now a long shot? *Ann. Neurol.* 85 303–315. 10.1002/ana.25410 30635926

[B31] Qian D. Jones C. Rzadzinska A. Mark S. Zhang X. Steel K. P. (2007). Wnt5a functions in planar cell polarity regulation in mice. *Dev. Biol.* 306 121–133. 10.1016/j.ydbio.2007.03.011 17433286 PMC1978180

[B32] Sellers K. J. Elliott C. Jackson J. Ghosh A. Ribe E. Rojo A. I. (2018). Amyloid beta synaptotoxicity is Wnt-PCP dependent and blocked by fasudil. *Alzheimers Dement.* 14 306–317. 10.1016/j.jalz.2017.09.008 29055813 PMC5869054

[B33] Sowers L. P. Mouw T. J. Ferguson P. J. Wemmie J. A. Mohapatra D. P. Bassuk A. G. (2013). The non-canonical Wnt ligand Wnt5a rescues morphological deficits in Prickle2-deficient hippocampal neurons. *Mol. Psychiatry* 18:1049. 10.1038/mp.2013.119 24056908

[B34] Styren S. D. Hamilton R. L. Styren G. C. Klunk W. E. (2000). X-34, a fluorescent derivative of Congo red: a novel histochemical stain for Alzheimer’s disease pathology. *J. Histochem. Cytochem.* 48 1223–1232. 10.1177/002215540004800906 10950879

[B35] Suzuki K. Iwata A. Iwatsubo T. (2017). The past, present, and future of disease-modifying therapies for Alzheimer’s disease. *Proc. Jpn. Acad. Ser. B Phys. Biol. Sci.* 93 757–771. 10.2183/pjab.93.048 29225305 PMC5790756

[B36] Tao H. Manak J. R. Sowers L. Mei X. Kiyonari H. Abe T. (2011). Mutations in prickle orthologs cause seizures in flies, mice, and humans. *Am. J. Hum. Genet.* 88 138–149. 10.1016/j.ajhg.2010.12.012 21276947 PMC3035715

[B37] Thal D. R. Walter J. Saido T. C. Fandrich M. (2015). Neuropathology and biochemistry of Abeta and its aggregates in Alzheimer’s disease. *Acta Neuropathol.* 129 167–182. 10.1007/s00401-014-1375-y 25534025

[B38] Tonnies E. Trushina E. (2017). Oxidative stress, synaptic dysfunction, and Alzheimer’s disease. *J. Alzheimers Dis.* 57 1105–1121. 10.3233/JAD-161088 28059794 PMC5409043

[B39] VanderVorst K. Dreyer C. A. Konopelski S. E. Lee H. Ho H. H. Carraway K.L. 3rd (2019). Wnt/PCP signaling contribution to carcinoma collective cell migration and metastasis. *Cancer Res.* 79 1719–1729. 10.1158/0008-5472.CAN-18-2757 30952630 PMC6467734

[B40] Veeman M. T. Axelrod J. D. Moon R. T. (2003). A second canon. Functions and mechanisms of beta-catenin-independent Wnt signaling. *Dev. Cell* 5 367–377. 10.1016/S1534-5807(03)00266-1 12967557

[B41] Wang Y. Guo N. Nathans J. (2006). The role of Frizzled3 and Frizzled6 in neural tube closure and in the planar polarity of inner-ear sensory hair cells. *J. Neurosci.* 26 2147–2156. 10.1523/JNEUROSCI.4698-05.2005 16495441 PMC6674805

[B42] Wang Z. Xiong L. Wan W. Duan L. Bai X. Zu H. (2017). Intranasal BMP9 ameliorates Alzheimer disease-like pathology and cognitive deficits in APP/PS1 transgenic mice. *Front. Mol. Neurosci.* 10:32. 10.3389/fnmol.2017.00032 28228716 PMC5296319

[B43] Whitaker R. Fossey J. Ballard C. Orrell M. Moniz-Cook E. Woods R. T. (2014). Improving well-being and health for people with Dementia (WHELD): study protocol for a randomised controlled trial. *Trials* 15 1745–6215. 10.1186/1745-6215-15-284 25016303 PMC4227075

[B44] Xu M. Zhang D. F. Luo R. Wu Y. Zhou H. Kong L. L. (2018). A systematic integrated analysis of brain expression profiles reveals YAP1 and other prioritized hub genes as important upstream regulators in Alzheimer’s disease. *Alzheimers Dement.* 14 215–229. 10.1016/j.jalz.2017.08.012 28923553

[B45] Yang Y. (2012). Wnt signaling in development and disease. *Cell Biosci.* 2:14. 10.1186/2045-3701-2-14 22520685 PMC3407699

[B46] Yarza R. Vela S. Solas M. Ramirez M. J. (2015). c-Jun N-terminal Kinase (JNK) signaling as a therapeutic target for Alzheimer’s disease. *Front. Pharmacol.* 6:321. 10.3389/fphar.2015.00321 26793112 PMC4709475

[B47] Zhang Q. Liu Y. Wang H. Ma L. Xia H. Niu J. (2017). The preventive effects of taurine on neural tube defects through the Wnt/PCP-Jnk-dependent pathway. *Amino Acids* 49 1633–1640. 10.1007/s00726-017-2462-x 28718066

[B48] Zhu W. Zhao L. Li T. Xu H. Ding Y. Cui G. (2019). Docosahexaenoic acid ameliorates traumatic brain injury involving JNK-mediated Tau phosphorylation signaling. *Neurosci. Res.* [Epub ahead of print]. 10.1016/j.neures.2019.07.008 31348997

