# Supplementary material for: Upregulation of Prickle2 Ameliorates Alzheimer’s Disease-Like Pathology in a Transgenic Mouse Model of Alzheimer’s Disease
Source: Front Cell Dev Biol. 2020 Sep 8;8:565020. doi: 10.3389/fcell.2020.565020 (PMC7509431; doi:10.3389/fcell.2020.565020)
Supplement: Supplementary file 1 [file Image_1.pdf]

Supplementary materials

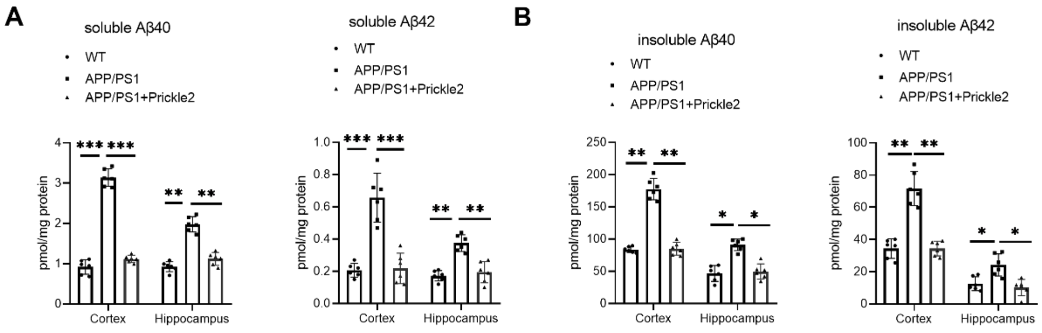

Fig. S1. ELISA assay was performed to measure the soluble and insoluble Aβs. (A) Soluble Aβs and (B) Insoluble Aβs were detected using ELISA (n = 6 per group). ANOVA followed by Bonferroni's post hoc test. *p < 0.05; **p < 0.01; ***p < 0.001; ****p < 0.0001.
